# Vaccine-induced SARS-CoV-2 antibody response: the comparability of S1-specific binding assays depends on epitope and isotype discrimination

**DOI:** 10.3389/fimmu.2023.1257265

**Published:** 2023-10-26

**Authors:** Silvia Schest, Claus Langer, Yuriko Stiegler, Bianca Karnuth, Jan Arends, Hugo Stiegler, Thomas Masetto, Christoph Peter, Matthias Grimmler

**Affiliations:** ^1^ Medizinisches Versorgungszentrum für Labormedizin und Mikrobiologie Ruhr GmbH, Essen, Germany; ^2^ Health University of Applied Sciences Tyrol, Innsbruck, Austria; ^3^ Institute of Molecular Medicine I, Heinrich Heine University Düsseldorf, Düsseldorf, Germany; ^4^ DiaSys Diagnostic Systems GmbH, Holzheim, Germany; ^5^ Institute for Biomolecular Research, Hochschule Fresenius gGmbH, University of Applied Sciences, Idstein, Germany; ^6^ DiaServe Laboratories GmbH, Iffeldorf, Germany

**Keywords:** SARS-CoV-2 antibody, spike protein, serological testing, COVID-19 vaccines, humoral immune response, neutralizing antibodies, WHO standard, correlate of protection

## Abstract

**Background:**

Quantification of the SARS-CoV-2-specific immune response by serological immunoassays is critical for the management of the COVID-19 pandemic. In particular, neutralizing antibody titers to the viral spike (S) protein have been proposed as a correlate of protection (CoP). The WHO established the First International Standard (WHO IS) for anti-SARS-CoV-2 immunoglobulin (Ig) (NIBSC 20/136) to harmonize binding assays with the same antigen specificity by assigning the same unitage in binding antibody units (BAU)/ml.

**Method:**

In this study, we analyzed the S1-specific antibody response in a cohort of healthcare workers in Germany (n = 76) during a three-dose vaccination course over 8.5 months. Subjects received either heterologous or homologous prime-boost vaccination with ChAdOx1 nCoV-19 (AstraZeneca) and BNT162b2 (Pfizer-BioNTech) or three doses of BNT162b2. Antibodies were quantified using three anti-S1 binding assays (ELISA, ECLIA, and PETIA) harmonized to the WHO IS. Serum levels of neutralizing antibodies were determined using a surrogate virus neutralization test (sVNT). Binding assays were compared using Spearman’s rank correlation and Passing–Bablok regression.

**Findings:**

All assays showed good correlation and similar antibody kinetics correlating with neutralizing potential. However, the assays show large proportional differences in BAU/ml. ECLIA and PETIA, which detect total antibodies against the receptor- binding domain (RBD) within the S1 subunit, interact similarly with the convalescent plasma-derived WHO IS but differently with vaccine serum, indicating a high sensitivity to the IgG/IgM/IgA ratio.

**Conclusion:**

All three binding assays allow monitoring of the antibody response in COVID-19-vaccinated individuals. However, the assay-specific differences hinder the definition of a common protective threshold in BAU/ml. Our results highlight the need for the thoughtful use of conversion factors and consideration of method-specific differences. To improve the management of future pandemics and harmonize total antibody assays, we should strive for reference material with a well-characterized Ig isotype composition.

## 1 Introduction

Coronavirus disease 2019 (COVID-19), caused by severe acute respiratory syndrome coronavirus 2 (SARS-CoV-2), is a global health challenge. Since the first case was reported in December 2019, the virus has spread rapidly to become a global pandemic, with more than 760 million confirmed cases and more than 6.9 million deaths worldwide as of May 2023 (1–3). Several countermeasures have been implemented, including the development of COVID-19 vaccines (4, 5).

The SARS-CoV-2 spike (S) protein is a primary target of neutralizing antibodies (nAbs), which are essential for protective immunity against viral infection (6–9). The receptor-binding domain (RBD), located in the S1 subunit of the trimeric S protein, mediates viral attachment by binding to the host cell receptor angiotensin-converting enzyme 2 (ACE2). The interaction between RBD and ACE2 plays a critical role in viral entry, making the SARS-CoV-2 S1 subunit a primary target for vaccine development (10–12).

The mRNA vaccine BNT162b2 (Pfizer BioNTech; hereafter referred to as BNT) and the vector vaccine ChAdOx1 nCoV-19 (Oxford-AstraZeneca; hereafter referred to as ChAd), were among the first COVID-19 vaccines authorized by the European Medicines Agency (EMA) between December 2020 and April 2021 (13, 14). Both vaccines, which encode the full-length S protein of SARS-CoV-2, have demonstrated high vaccine efficacy (VE) in clinical trials (11, 15–17).

Concerns about the durability of immunity and the ability of emerging SARS-CoV-2 variants of concern (VOCs) to evade immune protection have led to ongoing efforts to improve vaccination strategies. In December 2021, the World Health Organization (WHO) and EMA recommended the use of heterologous ‘prime-booster vaccination’ using different types of COVID-19 vaccines for the first and second doses; also known as the “mix-and-match” approach (18–20).

This decision was based on interim results from several clinical trials suggesting that heterologous vaccination results in a stronger and longer-lasting immune response. In particular, the combination of vector and mRNA vaccines appeared to induce higher levels of neutralizing antibodies than homologous vaccination with the same type of vaccine (21–26). In July 2022, the ECDC and EMA updated their public health recommendation, suggesting a second booster dose at least 4 months after the first (27). Those who received two doses of vector vaccine could receive a third dose of mRNA vaccine. Others received a homologous triple vaccination with three doses of mRNA vaccine, resulting in a heterogeneous vaccinated population.

Serological and cell-based assays are two common approaches used to quantify immune response and immune protection following vaccination (28, 29). While cell-based assays measure cellular immune responses such as T-cell proliferation or cytokine production, serological assays allow for the rapid and cost-effective quantification of SARS-CoV-2-specific antibodies in human serum. Therefore, serological assays are more suitable for routine diagnostics and high-throughput analysis in clinical laboratories. Serological tests can provide valuable information on VE and the durability of antibody protection, helping to identify individuals with suboptimal immune responses who may benefit from alternative vaccination strategies (30).

Neutralizing antibodies against the SARS-CoV-2 S protein are particularly important for assessing VE and predicting immune protection in individuals (31). High nAb titers have been associated with a lower risk of SARS-CoV-2 infection and severe COVID-19 disease. Several studies have shown that individuals with higher levels of neutralizing antibodies are less likely to develop symptomatic COVID-19 following natural infection or vaccination (32–36).

Neutralizing antibody titers have therefore been proposed as a correlate of protection (CoP) from SARS-CoV-2 (37). In a systematic review, Perry et al. found a robust correlation between vaccine-induced antibody levels and VE, despite the profound heterogeneity in vaccination regimens, serological assays, VE endpoints, and populations. The authors conclude that humoral immunity is an integral part of protection against COVID-19 and propose anti-S or neutralizing antibody levels as the most likely immune marker for a SARS-CoV-2 CoP (38).

In 2020, the WHO established the First International Standard (IS) for anti- SARS-CoV-2 immunoglobulin (NIBSC Code: 20/136) to harmonize serological test results worldwide (39–41). Reference standards are intended to improve the accuracy, reliability, and reproducibility of serological tests and facilitate the intercomparison of measurements obtained with different assays and detection methods in different laboratory settings worldwide (42). Lack of standardization can lead to the inaccurate interpretation of serological results, hampering effective disease surveillance and vaccine development (43).

In this study, we compared three SARS-CoV-2 S1-specific routine immunoassays for their ability to monitor humoral immune response and immune protection in a heterogeneous vaccination cohort. Subjects received different homologous and heterologous three-dose vaccination regimens over a period of 8.5 months. The serological tests, which differ in assay method (ELISA, ECLIA, and PETIA), antigens (full S1 subunit vs. RBD only), and isotype specificity (IgG vs. total Ig) were compared using Spearman’s rank correlation and Passing–Bablok regression. To define an universal cut-off for immune protection, suitable for real-world settings, we correlated anti-S1/RBD antibody titers (in BAU/ml) with neutralization potential (percentage inhibition of RBD-ACE2 interaction) as assessed by a surrogate virus neutralization test (sVNT).

## 2 Materials and methods

### 2.1 Study design and population

In this longitudinal observational study, we monitored the SARS-CoV-2 S1-specific antibody response in a cohort of healthcare workers in Germany who received three COVID-19 vaccinations (n = 76; median age, 50 years; interquartile range, 29–44 years; range, 23–68 years; female/male ratio, 6/1). Blood samples were collected at 11 fixed time points between February 2021 and January 2022. All participants were employed at the Medizinisches Versorgungszentrum für Labormedizin und Mikrobiologie Ruhr GmbH (mvzlm Ruhr) (Essen, Germany). Of the 80 subjects enrolled in this study, four participants were excluded from further analysis due to confirmed COVID-19 diagnosis (n = 2), pregnancy (n = 1), or allergic reaction (n = 1), resulting in a final study population of 76 eligible participants. This study was conducted in accordance with the World Medical Association’s Declaration of Helsinki and approved by the local ethics committee (Ärztekammer Nordrhein, No. 2021281). Participants gave written informed consent to participate in this study (44).

The majority of subjects received a homologous prime-boost vaccination with the vector vaccine ChAdOx1 nCoV-19 (Oxford-AstraZeneca; ChAd) as the first and second doses and the mRNA vaccine BNT162b (Pfizer BioNTech; BNT) as the third dose (63/76; 83%; ChAd-ChAd-BNT). The remaining subjects received either a heterologous prime-boost vaccination with ChAd as the first dose and BNT as the second and third doses (8/76; 11%; ChAd-BNT-BNT) or received a homologous vaccination with three doses of BNT (5/76; 7%; BNT-BNT-BNT).

Venous blood samples were collected at the following time points (TP): Before vaccination (TP1; -3/+ 0 days), 12 days (TP2; +/- 1 day) and 28 days after the first dose (TP3; +/- 2 days), the day of the second vaccination (TP4; -3/+0 days; administered 2.5 months after the first dose), 12 days (TP5; +/- 1 day), 28 days (TP6; +/- 2 days), 3 months (TP7; +/- 2 days) and 4 months after the second dose (TP8; +/- 2 days), the day of the third vaccination (TP9; +/- 2 days, administered 5 months after the second dose), and 12 days (TP10; +/- 1 day) and 28 days after the third dose (TP11; +/- 2 days).

Serum aliquots from collected blood samples were stored at −20°C until measurement. For unbiased comparison, an aliquot of each sample was thawed at room temperature and all serum samples for each time point were analyzed on all platforms on the same day, according to the manufacturer’s instructions.

### 2.2 Assays and instruments

#### 2.2.1 Anti-S1 immunoassays

Three different quantitative immunoassays were used to determine the serotiter of anti-SARS-CoV-2 antibodies specific for different proportions of the same S1-antigen (different epitope spectrum) (**Supplementary Table S1**). The Anti-SARS-CoV-2-QuantiVac IgG (Euroimmun, Lübeck, Germany) is an indirect enzyme-linked immunosorbent assay (ELISA) for the quantification of IgG antibodies against the complete S1 subunit of the SARS-CoV-2 S protein. Anti-SARS-CoV-2-QuantiVac IgG was performed on a fully automated Euroimmun Analyzer I (Euroimmun, Lübeck, Germany). The Elecsys^®^ Anti-SARS-CoV-2-S (Roche Diagnostics, Mannheim, Germany) is an electrochemiluminescence immunoassay (ECLIA) for the quantification of total antibodies (IgG, IgM, and IgA) against the RBD (located in the S1 subunit) in human serum and plasma. Elecsys^®^ Anti-SARS-CoV-2-S was performed on a fully automated e801 Cobas^®^ 8000 analyzer (Roche Diagnostics, Mannheim, Germany). The SARS-CoV-2 UTAB FS (Diasys Diagnostic Systems, Holzheim, Germany) is a particle-enhanced turbidimetric immunoassay (PETIA) for the quantification of total antibodies (IgG, IgA, and IgM) against the RBD in human serum and plasma. SARS-CoV-2 UTAB FS was performed on a fully automated c502 Cobas^®^ 8000 analyzer (Roche Diagnostics, Mannheim, Germany). Samples that exceeded linearity were measured in dilutions: SARS-CoV-2 UATB FS (range: 3.4–250 BAU/ml, dilutions: 1:20, 1:50, and 1:100); Elecsys^®^ Anti-SARS-CoV-2-S (range: 0.4–250 BAU/ml, dilutions: 1:20, 1:50, and 1:100); and Anti-SARS-CoV-2-QuantiVac IgG (range: 3.2–384 BAU/ml, dilutions: 1:10 and 1:100).

#### 2.2.2 Neutralization assay

The SARS-CoV-2 NeutraLISA (Euroimmun, Lübeck, Germany) is a semiquantitative competitive ELISA used as a surrogate virus neutralization test (sVNT; **Supplementary Table S1**). Neutralizing antibodies in the sample compete with the biotinylated ACE2 receptor in the sample buffer for binding to the precoated RBD. Bound ACE2 is detected by peroxidase-labeled streptavidin, which catalyzes a color reaction. The intensity of absorbance is inversely proportional to the concentration of neutralizing antibodies in the sample. Results are expressed as percentage inhibition (IH%) according to the following formula: IH% = 1 − (absorbance of sample/absorbance of blank) × 100. The negative cut-off is <20 IH% and the positive cut-off is ≥35 IH%. The SARS-CoV-2 NeutraLISA assay was performed on a fully automated Euroimmun-Analyzer I (Euroimmun, Lübeck, Germany).

#### 2.2.3 Harmonization to the WHO IS

All quantitative immunoassays have been harmonized using the First WHO International Standard (IS) for SARS-CoV-2 immunoglobulin (NIBSC code: 20/136) with an assigned unit of 250 International Units (IU) per vial for neutralizing activity. The final concentration after reconstitution is 1,000 IU/ml. Dilutions were 1:256, 1:128, 1:64, 1:32, 1:16, 1:8, and 1:4 (39, 41, 45). For ECLIA (1.0; Roche U/ml = BAU/ml) and ELISA (3.2; Euroimmun RU/ml x 3.2 = BAU/ml), conversion factors provided by the manufacturer were used. The conversion factor for PETIA (1.0; Diasys AU/ml = BAU/ml) was determined through calibration to the WHO IS (**Supplementary Figure S1**; **Supplementary Table S2**).

### 2.3 Statistical analysis

Spearman’s rank correlation and Passing–Bablok regression analysis (46, 47) were performed using MedCalc^®^ version 22.006 (MedCalc Software Ltd., Ostend, Belgium) according to the principles of CLSI Guideline C24 (48).

## 3 Results

### 3.1 Monitoring of vaccine-induced antibody response by anti-S1 binding assays

**Figure 1:**
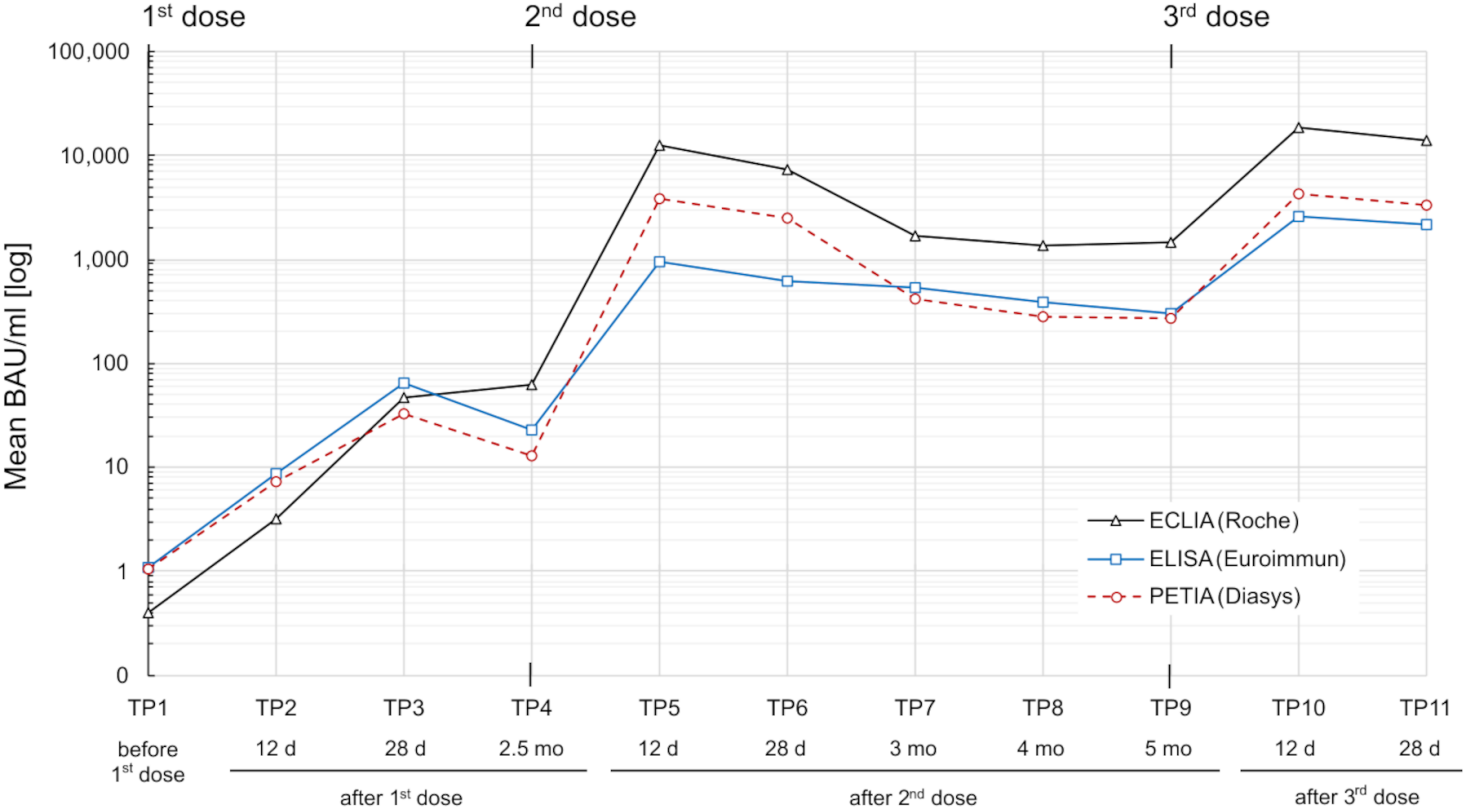
SARS-CoV-2 S1-specific antibody response in a heterogeneous vaccination cohort (n = 76) over 8.5 months. Serum samples were measured by three routine immunoassays: ECLIA (Roche; black line), ELISA (Euroimmun; blue line), and PETIA (Diasys; red dotted line). Mean binding antibody units per milliliter (BAU/ml) for each time point (TP1–TP11) are plotted in logarithmic scale.

Blood samples were collected at 11 different time points (TP1–TP11) during a three-dose COVID-19 vaccination course over 8.5 months. The cohort was vaccinated with ChAd-BNT-BNT, ChAd-ChAd-BNT, or BNT-BNT-BNT. S1-specific antibody serotiters were quantified using three routine binding assays and converted to BAU/ml: ELISA (Anti-SARS-CoV-2-QuantiVac IgG assay), ECLIA (Elecsys^®^ Anti-SARS-CoV-2-S assay), and PETIA (SARS-CoV-2 UTAB FS assay) (**Figure 1**).

The cohort displayed a heterogeneous antibody response with high interpatient variation in antibody titers (**Supplementary Figure S2**). Mean antibody levels increased rapidly after each vaccination, peaking at TP5 and TP10 for all three assays (**Figure 1**). The highest increases were observed 12 days after the second vaccination (from TP4 to TP5), ranging from 43-fold (ELISA) to 297-fold (PETIA), and 12 days after the third vaccination (from TP9 to TP10), ranging from 8-fold (ELISA) to 15-fold (PETIA) (**Table 1**; **Supplementary Figure S3**; **Supplementary Tables S3**-**S5**).

**Table 1:** SARS-CoV-2 S1-specific antibody titers (Mean BAU/ml).

Time point	TP1	TP2	TP3	TP4	TP5	TP6	TP7	TP8	TP9	TP10	TP11
**ECLIA**	0.4	3.2	46.5	62.7	12,704.4	7,410.1	1,705.0	1,379.8	1,456.9	1,8564.4	13,793.6
**ELISA**	0.4	8.5	61.0	21.8	959.7	582.5	522.6	373.7	284.0	2,601.9	1,766.5
**PETIA**	1.0	7.1	32.6	13.1	3,885.9	2,491.9	416.6	280.2	267.7	4,267.6	3,347.8
**Sample size**	68	68	62	59	62	63	59	53	57	54	41

Antibody levels began to decline as early as 28 days after the second vaccination (from TP5 to TP6) and 28 days after the third vaccination (from TP10 to TP11). Within 5 months after the first booster (from T5 to TP9), mean antibody titers had decreased to 30% (ELISA), 11% (ECLIA), and 7% (PETIA) of the peak concentration at TP5 (**Table 1**; **Supplementary Figure S4**).

Despite the similar kinetic profile, the mean BAU/ml values varied widely between the immunoassays, ranging from 959.7 BAU/ml (ELISA) to 12,704.4 BAU/ml (ECLIA) for TP5, and from 2,601.9 BAU/ml (ELISA) to 18,564.4 BAU/ml (ECLIA) for TP10 (**Table 1**). In general, the Elecsys^®^ Anti-SARS-CoV-2-S (ECLIA) assay measured consistently higher than the Anti-SARS-CoV-2-QuantiVac IgG (ELISA) or SARS-CoV-2 UTAB FS (PETIA) assays. The most considerable differences were observed in samples with the highest antibody titers. In these samples, the mean BAU/ml values for ECLIA were 13-fold (TP5/TP6) and 7-fold (TP10/TP11) higher than those for ELISA (**Table 1**; **Supplementary Figure S2**).

### 3.2 Comparison of anti-S1 binding assays

**Figure 2:**
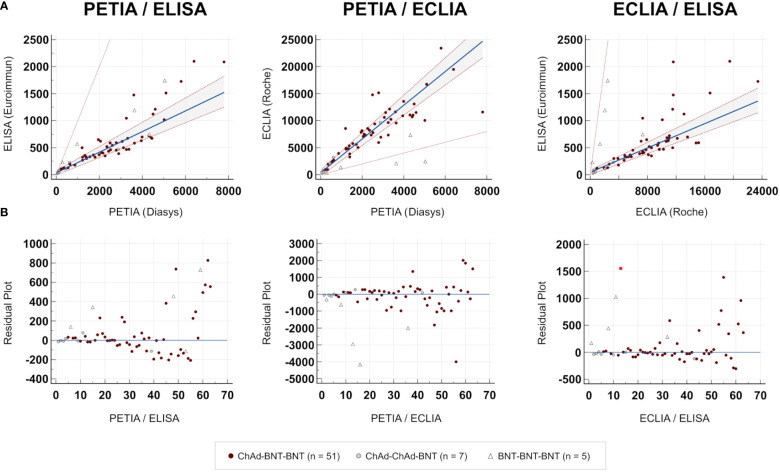
Comparison of immunoassays. Passing–Bablok regression analysis (TP6; n = 63). **(A)** Scatter diagram. Regression line (blue line), 95% CI of the regression line (dotted red lines), and identity line (thin red line). **(B)** Residual plot. Distribution of differences from the regression line (blue line). The red square indicates an outlier.

To further investigate these proportional differences, especially at high antibody titers, we compared all three assays through Passing–Bablok regression analysis. Slope and intercept were calculated with their respective 95% confidence intervals (CI), representing the systematic and proportional differences between the assays. Two methods can be considered to have no significant proportional differences if the 95% CI of the slope includes the value 1, e. g., slope = 1.01 (95% CI: 0.99–1.02). All three binding assays showed good overall correlation, with Spearman’s rank correlation coefficients (ρ) ranging from 0.77 (ELISA/ECLIA) to 0.92 (PETIA/ELISA) (**Figure 2**, **Table 2**). However, Passing–Bablok regression revealed significant proportional differences (deviation of slope from 1.00) between all three anti-S1 assays to varying degrees: 0.06 (ECLIA/ELISA; 95% CI: 0.05–0.07), 0.19 (PETIA/ELISA; 95% CI: 0.16–0.22), and 3.12 (PETIA/ECLIA; 95% CI: 2.80–3.47) (**Figure 2**, **Table 2**). The largest proportional difference was observed for ECLIA (anti-RBD) and ELISA (anti-S1), despite improvement by WHO harmonization (from 0.02 to 0.06; see **Supplementary Table S6**). Of note, the ELISA assay is specific for IgG antibodies, whereas ECLIA and PETIA do not discriminate by isotype, according to the manufacturers. Given the difference in antibody response after SARS-CoV-2 infection versus vaccination, this strongly suggests that calibration to the WHO IS does not improve the comparability of anti-S1 binding assays, especially if the assays are sensitive to differences in the IgG/IgM/IgA ratio.

**Table 2:** Passing–Bablok regression analysis (TP6).

	Spearman rank correlation	Passing–Bablok regression		Cusum test
	ρ (95% CI)	Intercept (95% CI)	Slope (95% CI)	P
**PETIA/ELISA**	0.92 (0.88–0.95)	34.50 (-7.67–71.60)	0.19 (0.16–0.22)	0.24
**PETIA/ECLIA**	0.82 (0.71–0.89)	367.74 (-200.60–830.25)	3.12 (2.80–3.47)	0.39
**ECLIA/ELISA**	0.77 (0.65–0.86)	45.73 (-0.70–79.77)	0.06 (0.05–0.07)	0.80

### 3.3 Correlation of antibody titers (BAU/ml) and neutralizing potential (sVNT IH%)

Next, we inquired whether we could still define a universal threshold in BAU/ml for all anti-S1 binding assays that correlate with humoral immune protection. Therefore, we analyzed the serum level of neutralizing anti-SARS-CoV-2 antibodies in each sample using a surrogate virus neutralization test (sVNT; SARS-CoV-2 NeutraLISA; Euroimmun).

**Figure 3:**
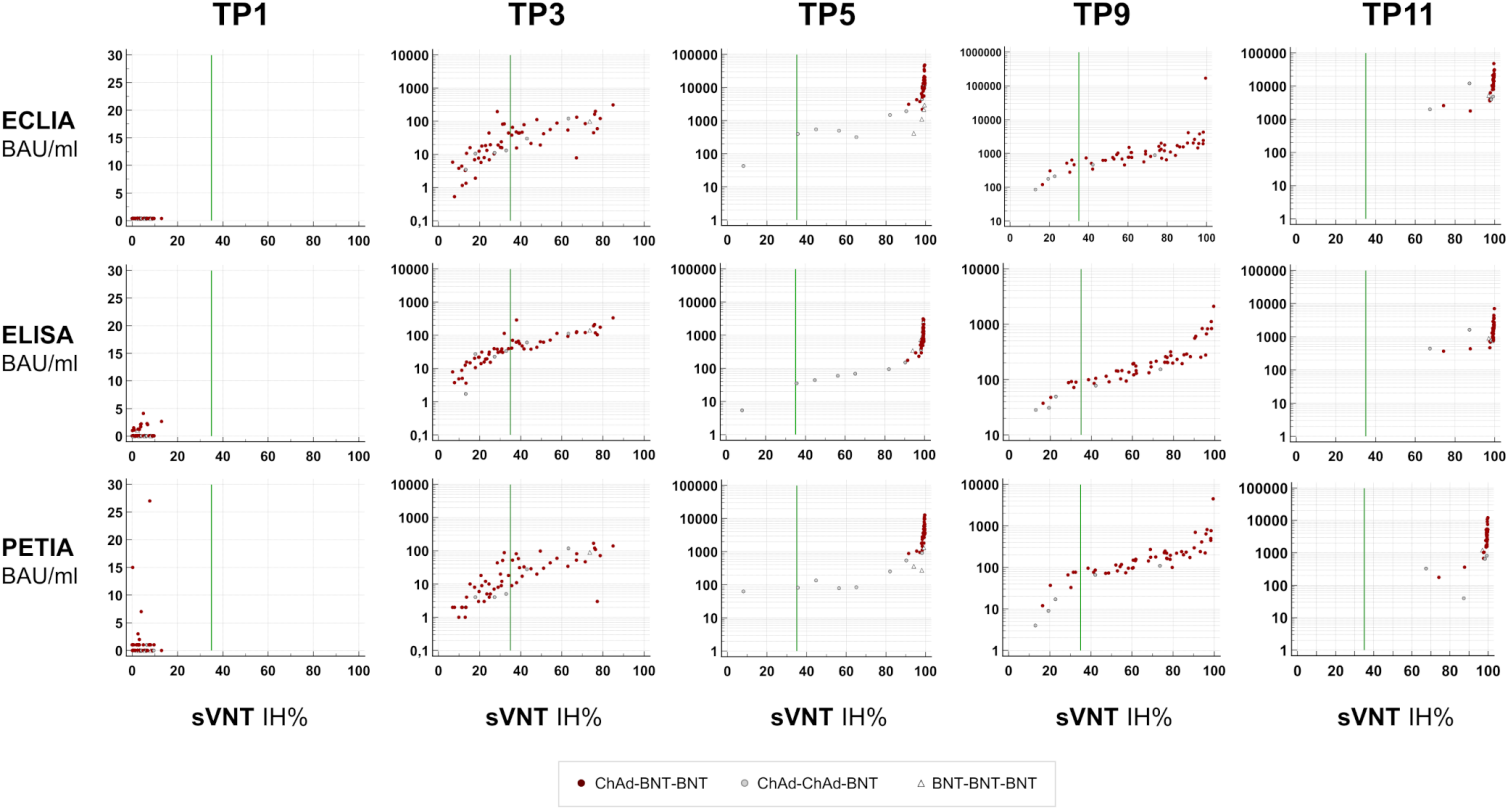
Neutralizing potential over time. S1-specific antibody titers (BAU/ml) for five selected time points are plotted against percentage inhibition (IH%) measured by a surrogate virus neutralization test (sVNT; SARS-CoV-2-NeutraLISA; Euroimmun). Negative cut-off, <20 IH%; positive cut-off, ≥35 IH% (green line).

**Figure 4:**
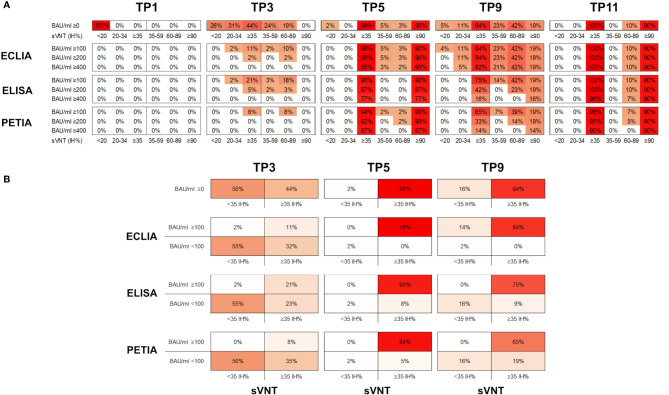
**(A)** Proportion of samples (%) in six different sVNT categories (<20; 20–45; ≥35, 35–59; 60–89; and ≥90 IH%) and above three potential BAU/ml thresholds (≥100; ≥200; and ≥400 BAU/ml) for five selected time points. **(B)** Proportion of samples (%) in four different categories (<35 IH%/<100 BAU/ml; <35 IH%/≥100 BAU/ml; ≥35 IH%/<100 BAU/ml; ≥35 IH%/≥100 BAU/ml) for three selected time points (TP3, TP5, and TP9).

In **Figure 3**, the percentage inhibition of RBD-ACE2 interaction (IH%) is plotted against the respective antibody titer at five selected time points (TP1, TP3, TP5, TP9, and TP11). In general, the kinetic of neutralizing potential paralleled the observed kinetic of antibody response; and both IH% and BAU/ml mean values peaked within 4 weeks after the second (TP5/TP6) and third (TP10/TP11) vaccinations, respectively (**Supplementary Figure S6**). The proportion of subjects above the positive sVNT cut-off (≥35 IH%) increased from 0% at TP1 to 98.4% at TP5 (12 days after the second dose; **Figure 4A**). At TP11 (28 days after the third dose), all subjects, regardless of vaccination schedule, had a neutralizing potential well above the positive cut-off (90% are ≥90 IH%, 100% are >60 IH%, **Figure 4A**; **Supplementary Figure S7**).

Interestingly, the decline in neutralizing potential did not parallel the waning in antibody titers after the second vaccination (from TP5 to TP9). While the mean IH% declined by 30% (from 93.8 to 64.4 IH%), the mean BAU/ml decreased more drastically during the same period: by 70% for ELISA, 89% for ECLIA, and 93% for PETIA (**Figure 4A**; **Supplementary Figure S4**). Furthermore, the proportion of subjects with neutralizing potential decreased substantially (from 90% of subjects >90 IH% at TP5 to 19% at TP5), but only 14% of the subjects fell below the positive neutralization cut-off at TP9 (from 98% of subjects ≥35 IH% to 84%) (**Figure 4B**). Despite the large relative change, the mean BAU/ml values did not fall below 100 BAU/ml at TP9 for all three assays, suggesting a potential threshold for immune protection (**Figure 1**; **Supplementary Figure S6**).

Given the heterogeneity of our cohort, we compared the kinetics of antibody titers and neutralizing potential by vaccination scheme (**Supplementary Figure S7**). Subjects receiving homologous prime-boost vaccination (ChAd-ChAd) had lower BAU/ml and IH% values after the second vaccination (TP5) than subjects receiving heterologous vaccination (ChAd-BNT). However, only one of the 76 subjects was clearly below the positive sVNT cut-off at TP5. The same subject was below 100 BAU/ml when measured by ECLIA (**Supplementary Figure S7**). Thus, for Elecsys^®^ Anti-SARS-CoV-2-S (Roche), antibody titers above 100 BAU/ml may indicate immune protection in our cohort. However, for ELISA and PETIA, 5% and 8% of all subjects were still below 100 BAU/ml at TP5, respectively, despite neutralizing potentials ≥35 IH% (**Figure 4A**; **Supplementary Figure S7**).

Five months after the second dose (TP9), 84% of all subjects were ≥35 IH% and all had antibody titers >100 BAU/ml as measured by ECLIA (**Figure 4B**). For ELISA and PETIA, only 75% and 65%, respectively, exceeded both thresholds. By contrast, the BAU/ml threshold failed to identify subjects without immune protection at TP9 for ECLIA: 16% of all subjects were <35 IH% but only 2% were <100 BAU/ml (**Figure 4B**). For ELISA and PETIA, the 100 BAU/ml threshold predicted subjects without immune protection (all subjects <35 IH% are <100 BAU/ml) but failed to identify subjects ≥35 IH% (i.e., not all subjects ≥35 IH% are >100 BAU/ml).

As seen at TP5, homologous prime-boost vaccination with ChAd-ChAd resulted in lower antibody titers and neutralizing potential than ChAd-BNT (**Figure S7**). Five months after the first booster (TP9), only 20% of the subjects vaccinated with ChAd-ChAd (4/5) showed antibody titers >100 BAU/ml, as measured by ELISA and PETIA. For ECLIA, 80% of subjects receiving ChAd-ChAd were >100 BAU/ml (100% for ChAd-BNT) (**Figure S7**). It is worth noting that we do not report significant differences between vaccination regimens due to the inherent limitations of our cohort. Therefore, we cannot draw any conclusions about the superiority of heterologous prime-boost vaccination with ChAd-BNT over BNT-BNT.

## 4 Discussion

In summary, our results indicate that all S1-specific binding assays facilitate monitoring of the antibody response in vaccinated individuals. All assays resulted in similar antibody kinetics, and increasing antibody titers were associated with increasing inhibitory potential. However, we were unable to define a clear cut-off value in BAU/ml across all methods that would help distinguish subjects above and below 35 IH%, mainly due to the large proportional differences between the binding assays.

The heterogeneity of vaccination schemes and individual immune responses adds another layer of complexity that further complicates the definition of a common threshold in BAU/ml. As previously reported, homologous prime-boost vaccination (ChAd-ChAd) appears to result in lower antibody titers and neutralizing potential than heterologous vaccination (ChAd-BNT). For example, 5 months after the second dose (TP9), all subjects are >100 BAU/ml for ECLIA, whereas all ChAd-ChAd vaccinated subjects are <100 BAU/ml when measured by PETIA, although both are anti-RBD total Ig assays that should correlate comparably with sVNT (**Supplementary Figure S7**). Interestingly, the substantial decline of S1-specific antibodies observed within 5 months after the first booster vaccination did not reflect a similar decline in inhibitory potential (sVNT IH%). It should be noted that neutralizing potential has been reported for anti-S1 antibodies raised against epitopes outside the RBD, whereas surrogate neutralization assays such as sVNT are limited to neutralizing anti-RBD antibodies. This may partially explain the observed differences in antibody waning between ECLIA/PETIA (anti-RBD) and ELISA (anti-S1) after the first booster.

The adaptive immune response to SARS-CoV-2 infection results in the activation and clonal expansion of virus-specific B cells. These differentiate into plasma cells and secrete soluble immunoglobins (Ig) into circulation that have different affinities for different viral proteins, mainly the viral spike (S) and nucleocapsid (N) protein in the case of SARS-CoV-2. Although pentameric IgM (low affinity/high avidity) provides the first line of defense, the subsequent seroconversion and production of high-affinity IgG are critical for long-term immune protection (49, 50). Neutralizing antibodies can inhibit the essential interaction between the RBD, located in the S1 subunit of the viral S protein, and the host cell receptor ACE2. Of note, IgA antibodies, which are responsible for mucosal immune defense, have been reported to exhibit ever higher neutralizing potential against SARS-CoV-2 than IgG antibodies (51). Immunization with vector or mRNA vaccines, on the other hand, results in S protein-specific antibodies raised against various epitopes in the S1 and S2 subunits.

The ability of different heterologous and homologous ChAd/BNT vaccination schemes to reduce SARS-CoV-2 infections or severe COVID-19 cases (VE) has been studied in large clinical trial populations (21–26, 52). A correlate of protection (CoP), on the other hand, is a measurable parameter that allows the prediction of immune protection in vaccinated individuals. Although spike-specific antibody titers have been proposed as a promising CoP for COVID-19, it is challenging to define which antibody titers are sufficient for immune protection (34, 37, 53). Several groups compared the antibody response in different ChAd/BNT vaccination cohorts using different routine binding assays (49, 54–56). These assays vary widely in antigen and isotype specificity as well as assay design and detection method. Spaeth et al. and Brehm et al. compared the performance of different N- and S-specific assays in SARS-CoV-2- positive subjects and patients with mild COVID-19 disease, respectively (57, 58). Here, we compared three anti-S1 binding assays in a heterogeneous vaccination cohort that use different parts of the same spike protein S1 subunit as antigen (RBD vs. full S1). It was not the aim of this study to compare the efficacy of different prime-boost vaccination regimens. However, it is worth noting that our results are consistent with previous reports, as homologous prime-boost vaccination with ChAd-ChAd seems to result in lower antibody titers than vaccination with ChAd-BNT or BNT-BNT (TP5). These differences are almost equalized after the third vaccination with BNT (TP11; **Supplementary Figure S7**) (59–63).

According to the WHO, an arbitrary unit of 1,000 BAU/ml can be used to assist the comparison of binding assays that detect “the same class of immunoglobulins with the same specificity” (45). Therefore, we asked ourselves the following question: How similar must anti-S1 binding assays be— in terms of isotype discrimination and assay principle— to meet this definition?

The ELISA assay (Euroimmun) detects IgG antibodies raised against the entire S1 subunit, whereas the ECLIA and PETIA assays both detect anti-RBD IgG, IgM, and IgA antibodies. Accordingly, PETIA and ECLIA show the smallest proportional difference, whereas ELISA and ECLIA show the lowest correlation and the largest proportional difference. Interestingly, PETIA and ECLIA interact similarly with the convalescent plasma-derived WHO IS (Roche U/ml = BAU/ml and Diasys AU/ml = BAU/ml) but yielded considerably different BAU/ml values in the heterogeneous vaccination cohort (**Supplementary Figure S1**, **Supplementary Table S2**) (39, 40). This might be explained by the difference in assay principles: in the Elecsys^®^ Anti-SARS-CoV-2 S assay (Roche), RBD-specific IgG, IgM, and IgA bind to a mix of biotinylated and ruthenylated RBD antigen. The resulting double-antigen sandwich (DAGS) complexes are immobilized on the solid phase via streptavidin-coated microparticles and quantified by electrochemiluminescence measurement. In the turbidimetric SARS-CoV-2 UTAB FS PETIA assay (Diasys), the RBD-antigen is coupled to polystyrene beads and binds to IgM, IgG, and IgA antibodies in the sample. The homogeneous PETIA assay can be more prone to non-specific reactions than heterogeneous technologies such as ELISA or (E)CLIA (64). Pentameric IgM tends to form larger antigen-antibody complexes than monomeric IgG or IgA and may result in a higher signal (65). In conclusion, the differential interaction of all three assays with the reference material, which is derived from SARS-CoV-2 infected individuals, versus vaccine serum strongly suggests that an alternative approach is required to harmonize different anti-S1 assays in a vaccination cohort.

The clinical benefits and intrinsic limitations of serological SARS-CoV-2-specific immunoassays are still vividly discussed (30, 66, 67). In particular, the repeated emergence of highly mutated VOCs, such as the *Omicron* variants, raised concerns that commercially available binding assays may become obsolete too quickly (66, 68, 69). More than thirty alterations have been identified within the *Omicron* spike protein, resulting in significantly reduced anti-RBD antibody binding and immune evasion (70–72). Wey et al. recently reported that the RBD-specific PETIA assay can quantify the antibody response to *Alpha* (B.1.1.7) and *Kappa* (B.1.617.1), while cross-reactivity to *Omicron* (B.1.1.529) is reduced by approximately 50% compared with wild-type virus and all other VOCs (64). In this study, we analyzed serum from subjects vaccinated in 2020/21, before the emergence of *Omicron* variants. We did not systematically compare the performance of all four binding assays in serum from patients infected with SARS-CoV-2 VOCs.

Other groups pointed out the inherent limitations of harmonization to the WHO IS, especially for SARS-CoV-2 binding assays that differ significantly in target antigen (N vs. S protein) and isotype specificity (IgG vs. IgM) (40, 59, 73–75). However, the early and widespread adoption of the WHO standard and the wide availability of conversion factors for commercial SARS-CoV-2 assays led to the following erroneous conclusion: conversion to BAU/ml allows the harmonization of two given SARS-CoV-2 binding assays. Of note, the WHO Expert Committee on Biological Standardization expressed concern that assigning the same unitage for binding assays based on different antigens would allow for an inappropriate use of the WHO IS (76). Our results confirm the distinct behavior of different anti-S1 binding assays: 1) assays that discriminate by isotype (IgG specific) but less by epitope (whole S1 subunit), and 2) assays that are more epitope specific (RBD only), but less isotype specific (total antibodies). Therefore, SARS-CoV-2 binding assays with the same antigen specificity and similar interaction with the WHO IS interact differently with vaccine serum. Furthermore, the correlation and proportional differences between ECLIA (heterogenous double-antigen sandwich assay) and PETIA (homogeneous turbidimetric assay) seem to change during the 8.5-month vaccination course, indicating a high susceptibility to the serum immunoglobulin composition (changing IgG/IgM/IgA ratio) (**Supplementary Figure S7**). This discrepancy, which is most likely due to different assay principles, adds another item to the list of hurdles we must overcome if we are to achieve proper harmonization of binding assay results, especially in populations that have received different vaccine regimens of varying efficacy.

Although all COVID-19 vaccines are based on the full-length S protein, the presentation of antigen-derived peptides is strikingly different —not only between protein-based and nucleic acid-based vaccines but also between mRNA (BNT) and vector (ChAd) vaccines. This in turn leads to different CD8+ and CD4+ T cell activation, which shapes the subsequent antibody response (77–80). It is conceivable that the heterogeneity of current vaccine platforms negatively affects the comparability of binding assays that detect total antibodies directed against the same antigen.

A viable way to overcome this limitation in the future would be to harmonize against the material of defined antigen specificity and/or isotype composition. Of note, Freeman et al. characterized five antigen-specific fractions of a serum-based reference material, containing antibodies against the SARS-CoV-2 S protein (anti-S1/S2, -S1, -S2, and anti-RBD) and N protein, for the standardization of IgG and total Ig serological assays (81). Consistent with our observations, the anti-RBD IgG assay (sCOVG) values were approximately the same for anti-RBD and anti-S1 sera, whereas the anti-RBD total antibody assay (COV2T) values were doubled for anti-S1 serum. Interestingly, the authors conclude that it is unlikely that IgM and IgA antibodies contribute to this discrepancy, as the serotiters of both have been reported to decline substantially by 6 weeks after symptom onset when sample collection for the reference material began (80). Nevertheless, the substantial discrepancy between the two anti-RBD total antibody assays, which varies over the course of vaccination in our cohort (**Supplementary Figure S7**), may still be due to differences in the two detection methods (PETIA vs. ECLIA).

The serum samples used for assay comparison were derived from a small non-representative cohort (n = 76) with a high female/male ratio and variable sample size per time point (n = 41–68; 32 subjects with ≥10 samples). Therefore, we do not report any significant difference between vaccination regimes, nor do we draw any conclusions about the superiority of heterologous prime-boost vaccination (ChAd-BNT vs. ChAd-ChAd). However, this assay comparison study has several limitations. Owing to the limited sample volume provided by the WHO, the 7-point WHO standard dilutions (3.9–250 BAU/ml) were assayed in singlets, which limits the accuracy of measurement. In addition, neutralizing antibodies were assessed by sVNT (inhibition of the RBD-ACE2 interaction), which does not reflect antiviral activity *in vivo*. However, surrogate assays are the only feasible way to estimate the neutralizing capacity of serum samples in clinical routine. The gold standard plaque reduction neutralization test (PRNT) is labor-intensive, time-consuming, and requires Biosafety Level 3 (BSL-3) facilities. Furthermore, sVNT is limited to neutralizing anti-RBD antibodies as detected by ECLIA and PETIA, whereas the ELISA assay detects antibodies against the entire S1 subunit (6–8).

Vaccine efficacy must be assessed using gold standard methods and studies must demonstrate a significant reduction in COVID-19 cases and/or severe disease progression in large study populations. However, in routine clinical practice, we must rely on cost-effective surrogate markers and surrogate assays to assess and evaluate the individual immune response in vaccinated individuals. Nevertheless, it is still under debate which marker(s) should be used for monitoring and what cut-off indicates adequate immune protection.

As discussed above, an *in vitro* CoP will never accurately predict vaccine efficacy and vaccine-induced immune protection in individuals, especially for highly evolving viruses, such as *Coronaviridae*. The recent COVID-19 pandemic has highlighted the need for rapid and flexible vaccine development and manufacturing, to ensure immune protection against emerging VOCs.

In summary, our results underscore the urgent need for rapidly evolving technology, not only for vaccines but also for serological binding assays, and for the continued development of both — bioanalytical methods and dedicated higher-order reference materials— to keep pace with rapidly mutating viruses. For future viral pandemics, if we are to use total antibody assays to monitor the vaccine-induced immune responses and predict immune protection in vaccinated individuals, we should strive to be more aware of method-specific differences and focus on the development of higher-order reference standards. Each reference material should be appropriate for the diagnostic task at hand, e.g., monitoring the antibody response post-infection versus post-vaccination.

## Data availability statement

The raw data supporting the conclusions of this article will be made available by the authors, without undue reservation.

## Ethics statement

The studies involving humans were approved by Ärztekammer Nordrhein, Tersteegenstr. 9, 40474 Düsseldorf, Germany. The studies were conducted in accordance with the local legislation and institutional requirements. The participants provided their written informed consent to participate in this study.

## Author contributions

SS: Formal Analysis, Writing – original draft, Writing – review & editing, Investigation, Methodology, Visualization. CL: Writing – review & editing, Conceptualization, Data curation, Supervision. YS: Conceptualization, Data curation, Supervision, Writing – review & editing, Resources. BK: Writing – review & editing, Investigation, Methodology. JA: Investigation, Methodology, Writing – review & editing. HS: Investigation, Methodology, Writing – review & editing, Validation. TM: Investigation, Validation, Writing – review & editing, Data curation, Formal Analysis. CP: Validation, Writing – review & editing, Resources. MG: Resources, Writing – review & editing, Conceptualization, Data curation, Formal Analysis, Supervision, Writing – original draft.
