# Supplementary material for: Vaccine-induced SARS-CoV-2 antibody response: the comparability of S1-specific binding assays depends on epitope and isotype discrimination
Source: Front Immunol. 2023 Oct 26;14:1257265. doi: 10.3389/fimmu.2023.1257265 (PMC10641008; doi:10.3389/fimmu.2023.1257265)
Supplement: Supplementary file 1 [file DataSheet_1.pdf]

Supplementary Material

Vaccine-induced SARS-CoV-2 antibody response: the comparability of S1-specific binding assays depends on epitope and isotype discrimination

Silvia Schest, Claus Langer, Yuriko Stiegler, Bianca Karnuth, Jan Arends, Hugo Stiegler, Thomas Masetto, Christoph Peter, Matthias Grimmler

1 Supplementary Figures

Supplementary Figure S1. Standard curve. First WHO International Standard for SARS-CoV-2 immunoglobulin (NIBSC 20/136; Dilutions: 1:256, 1:128, 1:64, 1:32, 1:16, 1:8, 1:4) measured by ELISA (Euroimmun), ECLIA (Roche), and PETIA (DiaSys). Regression line (blue line), 95% CI of the regression line (dotted red lines), and identity line (thin red line).

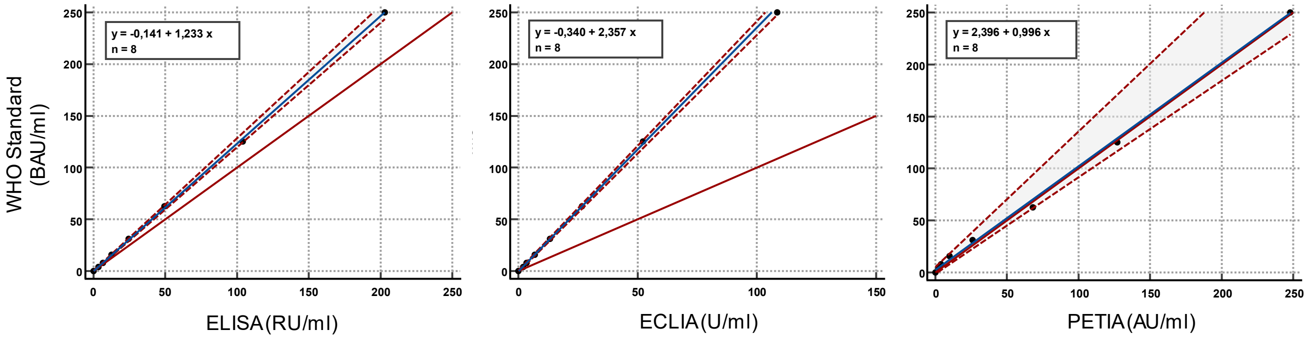

Supplementary Figure S2. Box-and-Whisker plots of BAU/ml values for each time point (TP1–TP11) measured by ECLIA (Roche), ELISA (Euroimmun), and PETIA (DiaSys). Box-plots show median, interquartile range (IQR) and upper/lower adjacent value (1.5 times outside IQR). Outliers (3 times outside IQR) are represented as red squares.

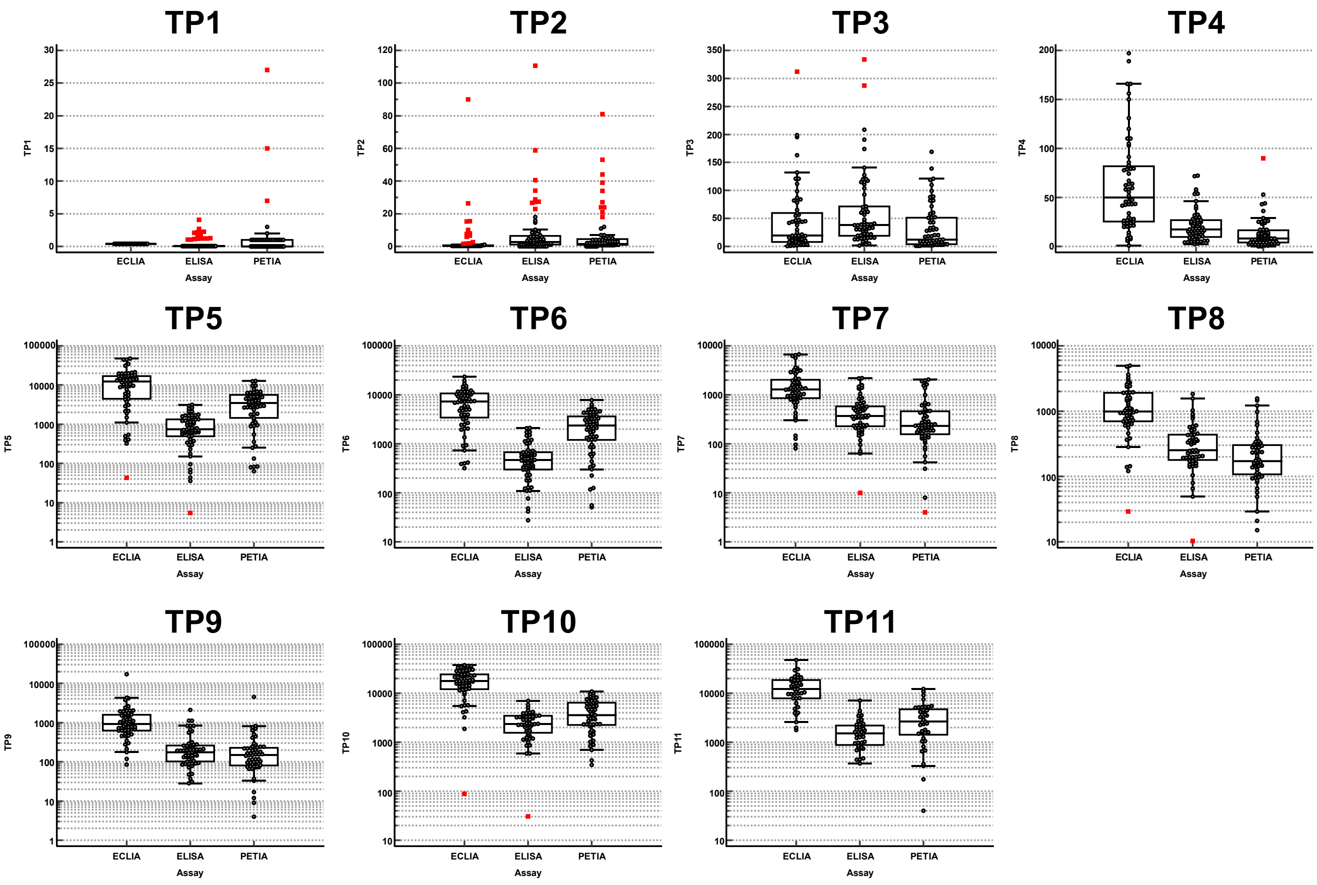

Supplementary Figure S3. Antibody response. Mean BAU/ml values (<u>non</u>-log scale) measured by ECLIA (Roche; black line), ELISA (Euroimmun; blue line), and PETIA (DiaSys; red dotted line).

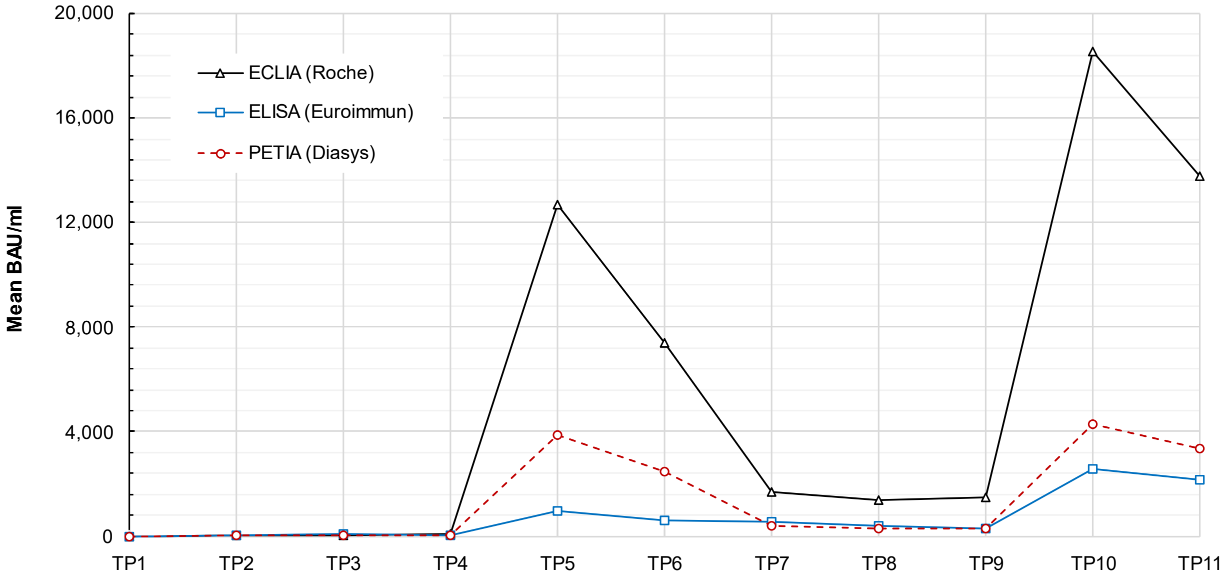

Supplementary Figure S4. Passing-Bablok regression analysis. Reduction of proportional differences (slope) after conversion to BAU/ml for ELISA (Euroimmun). Conversion factor for ECLIA and PETIA is 1.0 (AU/ml = BAU/ml; U/ml = BAU/ml).

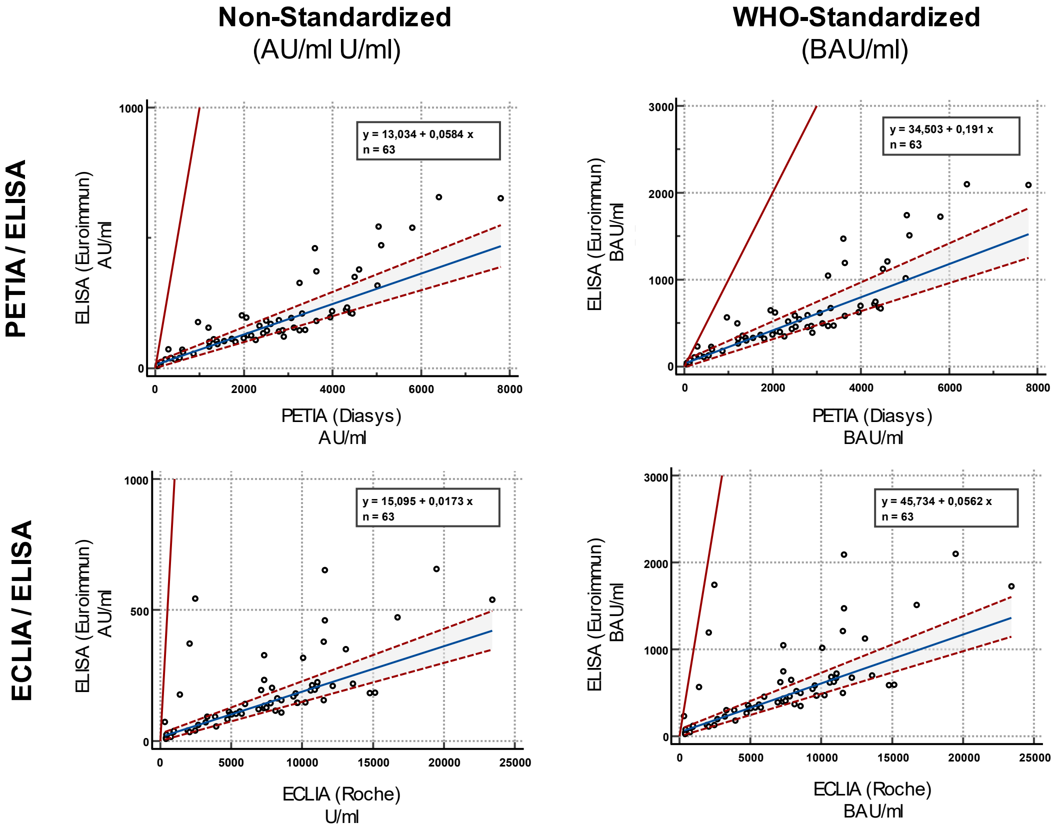

Supplementary Figure S5. Percent change in BAU/ml (log scale) measured by ECLIA (Roche; black line), ELISA (Euroimmun; blue line), and PETIA (DiaSys; red dotted line), versus change in neutralizing potential (sVNT IH%) after the second vaccination (TP5–TP1). Mean values are normalized to TP5.

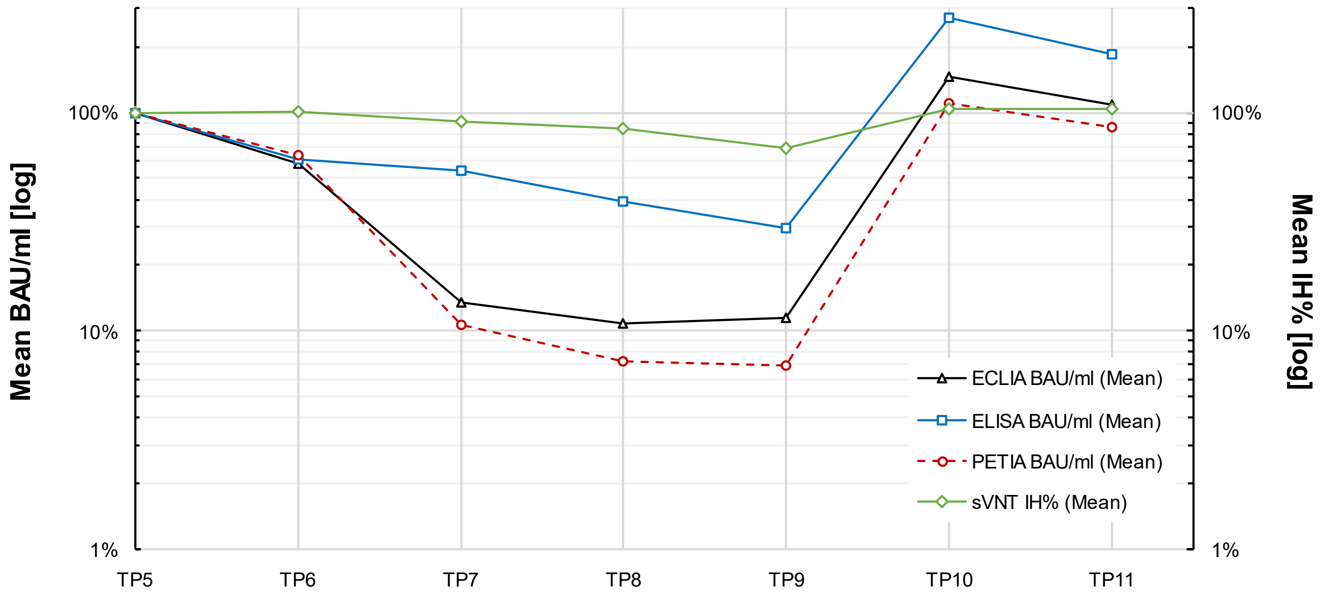

Supplementary Figure S6. Antibody response (Mean BAU/ml; log scale) measured by ECLIA (Roche; black line), ELISA (Euroimmun; blue line), and PETIA (DiaSys; red dotted line), compared to the neutralizing potential (sVNT IH%) over time.

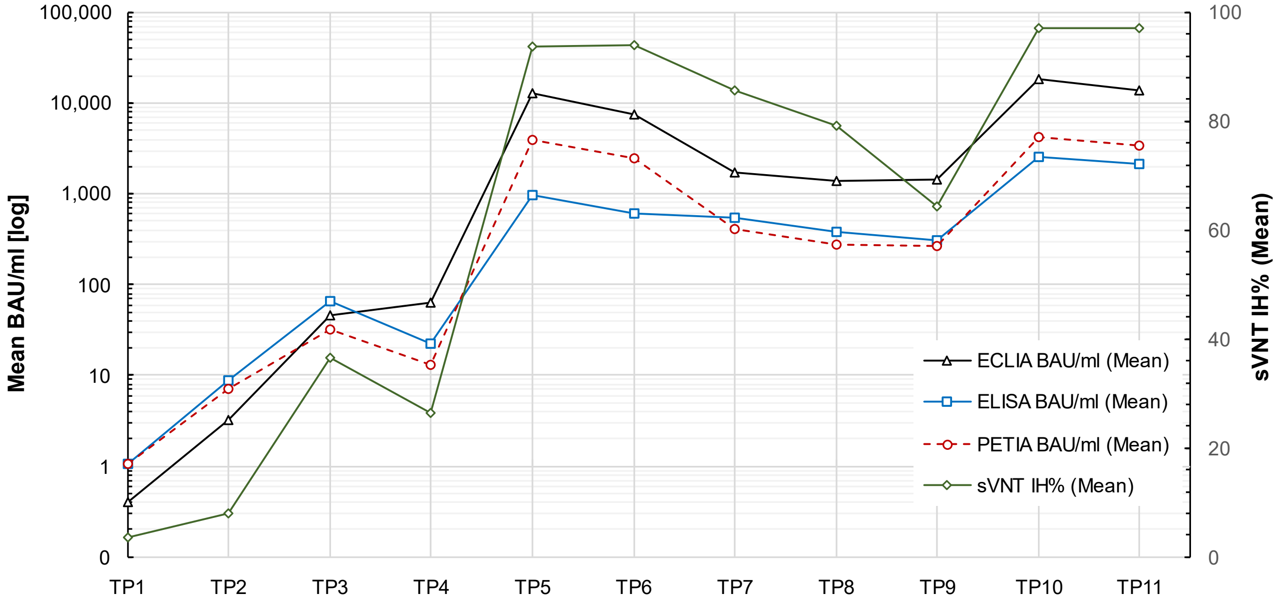

Supplementary Figure S7. Subgroup analysis by vaccination scheme. Box-and-Whisker plots of neutralizing potential (sVNT IH%) and antibody titers (BAU/ml) measured by ECLIA (Roche), ELISA (Euroimmun), and PETIA (DiaSys) for five selected time points. ChAd-BNT-BNT (red dots), ChAd-ChAd-BNT (grey dots), BNT-BNT-BNT (white triangles). Box-plots show median, interquartile range (IQR) and upper/lower adjacent value (1.5 times outside IQR). Outliers (3 times outside IQR) are represented as red squares. sVNT cut-off (≥35 IH%; green line), potential BAU/ml threshold (>100 BAU/ml, blue line).

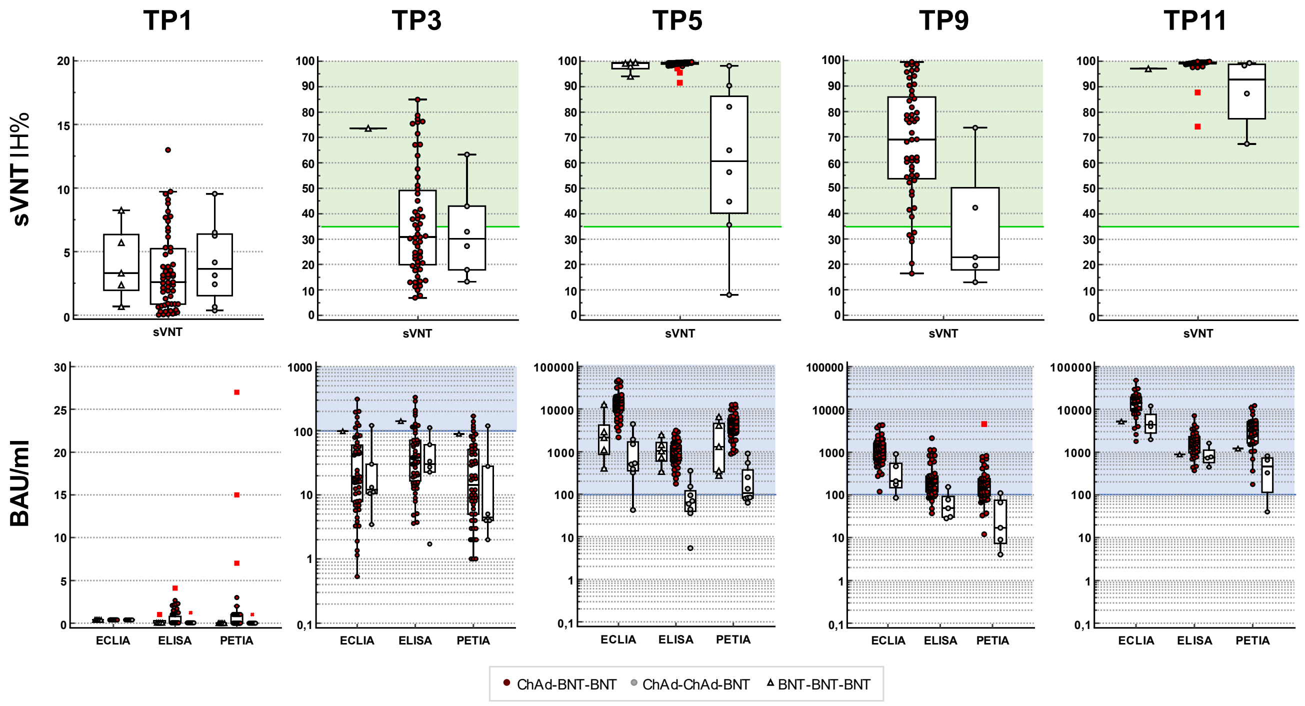

2 Supplementary Tables

Table S1. Binding Assay Characteristics

**Assay**	**Elecsys®<br>Anti-SARS-CoV-2 S**	**SARS-CoV-2<br>UTAB FS**	**Anti-SARS-CoV-2<br>QuantiVac IgG**
**Manufacturer**	Roche Diagnostics (Mannheim, Germany)	DiaSys Diagnostic Systems (Holzheim, Germany)	Euroimmun (Lübeck, Germany)
**Method**	Double antigen sandwich electro-chemiluminescence immunoassay (ECLIA)	Particle enhanced immunoturbidimetric assay (PETIA)	Enzyme-linked immunosorbent*t assay (ELISA)
**Immunoglobulin**	IgG / IgM / IgA	IgG / IgM / IgA	IgG
**Antigen**	Receptor binding domain (RBD) within S1 subunit	Receptor binding domain (RBD) within S1 subunit	Full-length S1 subunit
**Quantification**	quantitative	quantitative	quantitative
**Calibration**	2-point	5-point	6-point
**Range**	0.4–250 BAU/ml	1.5–150 BAU/ml	3.2–384 BAU/ml
**Conversion factor**	U/ml = 1.0 BAU/ml	AU/ml = 1.0 BAU/ml	RU/ml = 3.2 BAU/ml

Table S2. WHO Standardization and BAU/ml Conversion Factors.

**Dilution**	**1:256**	**1:128**	**1:64**	**1:32**	**1:16**	**1:8**	**1:4**	**Conversion factor (Slope Fig. S3)**	**Conversion factor (Manufacturer)**
**WHO (BAU/ml)**	3.9	7.8	15.6	31.3	62.5	125.0	250.0		
**ECLIA (U/ml)**	3.4	6.6	12.5	24.4	49.4	104.0	203.0	1.2	**1.0**
**ELISA (RU/ml)**	2.0	3.5	6.9	13.3	26.7	52.3	108.5	2.4	**3.2**
**PETIA (AU/ml)**	2.0	4.0	10.0	26.0	68.0	127.0	248.0	**1.0**	-

Table S3. ECLIA (Elecsys® Anti-SARS-CoV-2 S; Roche)

	**TP1**	**TP2**	**TP3**	**TP4**	**TP5**	**TP6**	**TP7**	**TP8**	**TP9**	**TP10**	**TP11**
**Mean BAU/ml**	0.4	3.2	46.5	62.7	12704.4	7410.1	1705.0	1379.8	1456.9	18564.4	13793.6
**SD**	0.0	11.5	58.4	48.2	10541.1	4907.3	1508.4	1096.8	2287.4	9637.4	9029.3
**Q1 (25%)**	0.4	0.4	8.6	25.7	4616.8	3582.0	859.5	701.0	628.0	12166.3	7860.0
**Q2 (50%) / Median**	0.4	0.4	19.3	49.9	12217.0	7326.0	1294.0	992.0	918.0	24204.3	18484.0
**Q3 (75%)**	0.4	0.7	58.9	81.1	16876.3	10655.5	2003.5	1903.0	1572.0	17821.0	12226.0
**Maximum BAU/ml**	0.4	90.0	312.0	197.0	47572.0	23408.0	6694.0	4950.0	17028.0	37562.0	47675.0
**Sample size**	68	68	62	59	62	63	59	53	57	54	41

Table S4. ELISA (Anti-SARS-CoV-2 QuantiVac IgG; Euroimmun)

	TP1	TP2	TP3	TP4	TP5	TP6	TP7	TP8	TP9	TP10	TP11
**Mean BAU/ml**	0.4	8.5	61.0	21.8	959.7	582.5	522.6	373.7	284.0	2601.9	1766.5
**SD**	0.8	16.7	66.6	16.7	719.2	480.2	465.9	342.7	352.9	1366.1	1288.4
**Q1 (25%)**	0.0	1.2	19.1	9.8	496.3	299.1	230.4	181.3	103.1	1592.2	890.3
**Q2 (50%) / Median**	0.1	2.7	38.1	17.4	744.5	467.1	370.4	252.3	175.3	2362.9	1519.8
**Q3 (75%)**	0.0	6.2	71.2	26.7	1327.2	672.7	578.4	435.4	257.8	3431.7	2162.9
**Maximum BAU/ml**	4.1	110.7	334.0	72.1	3124.0	2100.0	2185.6	1840.9	2106.3	6991.2	7081.1
**Sample size**	68	68	62	59	62	63	59	53	57	54	41

Table S5. PETIA (SARS-CoV-2 UTAB FS; DiaSys)

	TP1	TP2	TP3	TP4	TP5	TP6	TP7	TP8	TP9	TP10	TP11
**Mean BAU/ml**	1.0	7.1	32.6	13.1	3885.9	2491.9	416.6	280.2	267.7	4267.6	3347.8
**SD**	3.8	14.3	39.8	15.4	3002.6	1734.8	494.0	330.3	598.4	2785.3	2787.9
**Q1 (25%)**	0.0	1.0	4.0	4.0	1527.8	1207.5	162.5	106.0	82.0	2262.5	1500.0
**Q2 (50%) / Median**	0.1	1.5	12.0	8.0	3486.0	2352.0	233.0	168.0	150.0	3560.5	2650.0
**Q3 (75%)**	1.0	4.3	50.0	16.0	5475.0	3616.5	455.5	294.0	226.0	6400.0	4575.0
**Maximum BAU/ml**	27.0	81.0	169.0	90.0	12800.0	7800.0	2065.0	1561.0	4500.0	10900.0	12250.0
**Sample size**	68	68	62	59	62	63	59	53	57	54	41

Table S6. Impact of harmonization to the WHO IS (TP6).

	**Spearman Correlation ρ (95% CI)**		**Proportional Difference Slope (95% CI)**	
	**AU/ml / RU/ml U/ml**	**BAU/ml**	**AU/ml / RU/ml U/ml**	**BAU/mll**
**PETIA / ELISA**	0.92 (0.88–0.95)	0.92 (0.88–0.95)	**0.06** (0.05-0.07)	**0.19** (0.16–0.22)
**ECLIA / ELISA**	0.77 (0.65–0.86)	0.77 (0.65–0.86)	**0.02** (0.01-0.02)	**0.06** (0.05–0.07)
**PETIA / ECLIA**	0.82 (0.71–0.89)	0.82 (0.71–0.89)	3.12 (2.80–3.47)	3.12 (2.80–3.47)

Table S7. Proportional Difference and Spearman Rank Correlation (ρ) over time (PETIA/ECLIA).

	TP3	TP4	TP5	TP6	TP7	TP8	TP9	TP10	TP11
**Slope** (95% CI)	1.54 (1.30–1.81)	4.89 (4.23–5.77)	3.28 (2.97–3.63)	3.12 (2.80–3.47)	4.40 (3.89–5.05)	5.79 (5.13–6.70)	6.57 (5.76–7.83)	4.21 (3.67–5.03)	3.85 (3.44–4.25)
**Correlation** **ρ** (95% CI)	0.83 (0.74– 0.90)	0.89 (0.82–0.93)	0.89 (0.82–0.93)	0.82 (0.71–0.89)	0.91 (0.86–0.95)	0.91 (0.85–0.95)	0.92 (0.87–0.95)	0.86 (0.78–0.92)	0.90 (0.82–0.95)
